# Wireless Patch Antenna Characterization for Live Health Monitoring Using Machine Learning

**DOI:** 10.3390/s25154654

**Published:** 2025-07-27

**Authors:** Dominic Benintendi, Kevin M. Tennant, Edward M. Sabolsky, Jay Wilhelm

**Affiliations:** 1 School of Electrical Engineering and Computer Science, Russ College of Engineering and Technology, Ohio University, Athens, OH 45701, USA; db821617@ohio.edu; 2 Department of Mechanical, Materials and Aerospace Engineering, West Virginia University, Morgantown, WV 26506, USA; kmt0007@mix.wvu.edu (K.M.T.); ed.sabolsky@mail.wvu.edu (E.M.S.); 3 Mechanical Engineering Department, Russ College of Engineering and Technology, Ohio University, Athens, OH 45701, USA

**Keywords:** Vector Network Analyzer, Long Short-Term Memory, wireless interrogation, patch antenna, temperature estimation, machine learning, far-field sensing

## Abstract

Temperature monitoring in extreme environments, such as coal-fired power plants, was addressed by designing and testing wireless patch antennas for use in machine learning-aided temperature estimation. The sensors were designed to monitor the temperature and health of boiler systems. Wireless interrogation of the sensor was performed using a Vector Network Analyzer (VNA) and a pair of interrogation antennas to capture resonance behavior under varying thermal and spatial conditions with sensitivities ranging from 0.052 to 0.20 $\frac{MHz}{{}^{\circ}C}$. Sensor calibration was conducted using a Long Short-Term Memory (LSTM) model, which leveraged temporal patterns to account for hysteresis effects. The calibration method demonstrated improved performance when combined with an LSTM model, achieving up to a 76% improvement in temperature estimation error when compared with Linear Regression (LR). The experiments highlighted an innovative solution for patch antenna-based non-contact temperature measurement, which addresses limitations with conventional methods such as RFID-based systems, infrared, and thermocouples.

## 1. Introduction

Monitoring machinery temperatures in extreme environments requires sensing systems capable of withstanding harsh conditions. Passive wireless Radio Frequency (RF) based temperature sensors have been developed to address these issues, where calibration and accuracy could be improved [1,2]. RF patch antennas can be designed to resonate at a specific frequency that changes with temperature, allowing remote measurements [1,2,3,4,5,6,7,8,9,10,11,12,13,14,15,16]. Sensitivity variation, calibration, and hysteresis in patch antennas remain underexplored, limiting their deployment and accuracy. Live data processing from RF patch antenna sensors will be needed to enable real-time monitoring. The goal of this work was to develop an accurate RF patch antenna-based temperature monitoring process that uses Machine Learning (ML) for calibration.

Temperature monitoring using RF patch antenna sensors requires a contactless interrogation system capable of operation at far-field ranges [1,3,4,5,7,8,9,11,12,13,14,15,16]. Measurement of reflected RF energy can be accomplished using a Vector Network Analyzer (VNA), which will wirelessly interrogate a sensor to capture its resonance behavior under varying thermal and spatial conditions. Contactless operation is made possible by this setup, eliminating the need for wired connections in high-temperature environments. The operating principle of patch antenna sensors lies in the temperature dependence of their resonant frequency, which shifts as the material properties and geometry of the antenna are altered by thermal conditions. Measuring this relationship requires controlled heating of the sensor while recording its resonance response. Sensor data will be collected by heating the sensor across the full temperature range of an oven, from 20 °C to 170 °C, to simulate temperature-induced shifts in resonant frequency under conditions representative of boiler environments. Interrogation distances are of interest to evaluate how signal strength and sensitivity degrade with range, allowing the system to be calibrated for far-field wireless operation. Time-gating can further improve received signals by isolating areas of interest and suppressing interference from multipath reflections, improving feature quality for ML [17]. Calibration of patch antenna sensors can utilize a Long Short-Term Memory (LSTM) model that leverages temporal patterns and reduces hysteresis. The effectiveness of the developed sensor reading system will be validated using Mean Absolute Error (MAE) and an $R^{2}$ score with comparisons to traditional regression methods. The automated calibration method will enable temperature predictions without the need for post-processing data. The combined calibration and sensor reading system is presented in this paper, with results describing functionality and performance.

Traditional approaches to in situ temperature monitoring, such as thermocouples, rely on wired connections that are susceptible to wear in high-temperature environments [9,18,19,20]. Radio Frequency Identification (RFID) systems eliminate wiring but still depend on onboard electronics, which can degrade with temperatures above 250 °C [2,7]. Optical solutions such as infrared (IR) sensors require consistent surface emissivity for accurate temperature readings, as variations caused by oxidation, soot buildup, or material changes can introduce errors of up to 136 °C when emissivity deviates by 10% [9,21,22]. Current industry standards for estimating boiler temperatures, such as acoustic pyrometers, infer temperature indirectly by analyzing the acoustic properties of flue gases, rather than performing direct measurements of the boiler structure itself [23]. Passive systems do not require physical wiring, unlike active sensors, making them well-suited for monitoring in harsh environments where access is limited. Loop antennas offer a similarly passive alternative and support circular polarization [24,25], but are limited to near-field operation, restricting long-range use. Surface acoustic wave (SAW) sensors also provide fully passive temperature sensing by reflecting radio signals from a piezoelectric substrate, although they require precise fabrication [26,27,28]. RF patch antennas are a type of passive sensor and are commonly rectangular or circular in shape. Each sensor includes a conductive patch, often made of copper, placed on top of a grounded dielectric substrate and mounted to a flat surface. The metallic patch functions as the radiating element, while the ground plane directs the signal [29]. Patch antennas are preferred for their ease of fabrication, flat form factor, and suitability for installation in confined spaces, and were chosen over high-Q alternatives due to their low profile and robustness in high-temperature environments, even though high-Q antennas offer sharper resonance shifts.

Patch antennas were first investigated for use in structural health monitoring (SHM) as passive wireless sensors whose resonant frequency would shift in response to structural degradation [30,31,32,33,34,35,36,37,38]. Pressure detection [39] and temperature monitoring [1] are also possible by examining how a patch antenna’s resonant frequency shifts [1,3,4,5,7,8,9,11,12,13,14,15,16]. Interrogation setups typically involve a single interrogation antenna ($S_{11}$) at near-field ranges [9,11,12,13,14,15], two interrogation antennas ($S_{21}$) at far-field ranges [1,4,8,16], or a wired connection to the sensor via an impedance-matched feedline [3,5,7]. Received antenna data is typically recorded using a VNA that can output a frequency sweep and measure received power and phase [1,3,4,5,7,8,9,11,12,13,14,15,16]. Patch antenna research has not developed algorithms for accurate temperature estimation based on these measurements, nor has it accounted for the effect of hysteresis on sensor calibration. Hysteresis was typically mitigated by sensor stabilization or slow thermal cycling prior to VNA readings, but such controlled and delayed conditions are impractical for continuous, live temperature monitoring. Recent research has not explored how other aspects of the resonance response, such as resonant magnitude and phase, could contribute to temperature estimation [8], which suggests the need for a temperature estimation system that incorporates additional trainable variables as model features to improve sensor calibration and prediction accuracy. Few studies have varied the distance between the interrogator antennas and the sensor. Notable exceptions include Tennant et al. [1] and Ma et al. [13], though their conclusions are based on a limited number of experiments, reducing the generalizability of their results. Post-processing of $S_{21}$ data was typically performed using MATLAB or LabVIEW [1,3,4,5,7,8,9,11,12,13,14,15,16], but these tools lack the hardware compatibility and live performance needed for this work. Enabling sensor calibration for use in live temperature monitoring required a unified program that directly interfaces with all hardware.

Accurate, real-time temperature estimation in the presence of hysteresis and temporal variability requires an alternative to traditional calibration methods. Temporal models capable of learning sequential trends from past data offer a viable solution. Long Short-Term Memory networks (LSTMs) are a type of recurrent neural network that overcomes the vanishing gradient problem in traditional RNNs and are widely used for time series prediction [40,41,42,43]. LSTMs achieve proficiency in temporal predictions by employing a memory cell and three gates: the forget gate (deciding what information to discard), the input gate (determining what new information to store), and the output gate (controlling what information to output). LSTMs are designed to selectively retain or discard information across *N* timesteps, making them particularly effective for tasks such as time-series prediction [40,41,42,43]. Precautions are usually taken to avoid overfitting or underfitting an LSTM model. Overfitting occurs when a model learns too much from training data, harming its performance on unseen data, while underfitting results from insufficient learning of data patterns.

## 2. Materials and Methods

Developing a live, machine learning–calibrated patch antenna temperature monitoring system required the fabrication of a sensor with a known resonant frequency, along with hardware for data acquisition, a signal isolation process, data for model training, and live inference. High-temperature boiler systems require passive sensors that can operate without embedded electronics or wired connections. Patch antennas fabricated from ceramic dielectric substrates and heat-resistant metals provide a robust solution capable of withstanding extreme environments [1,44]. Patch antennas were selected due to their passive sensing in harsh environments, compact form factor, and ease of fabrication. Determining the sensor’s temperature from its resonance response required interrogator antennas, a Vector Network Analyzer (VNA), and signal processing. The sensor system was tested with 28 datasets across five interrogation distances (200–900 mm) under both rising and falling temperature conditions using two patch antenna variants with the same resonant frequency, enabling the system to capture thermal and spatial variation necessary for model generalization. These two sensors were fabricated using identical geometry and material composition; the second unit was included to verify consistency and reproducibility of the sensor response and ensure that performance was not specific to a single device. Data collected from the system was used to train the machine learning models, which could be used for real-time analysis. The patch antenna design process, experimental setup, ML training, and signal analysis are described in this section.

### 2.1. Patch Antenna Design

**Figure 1:**
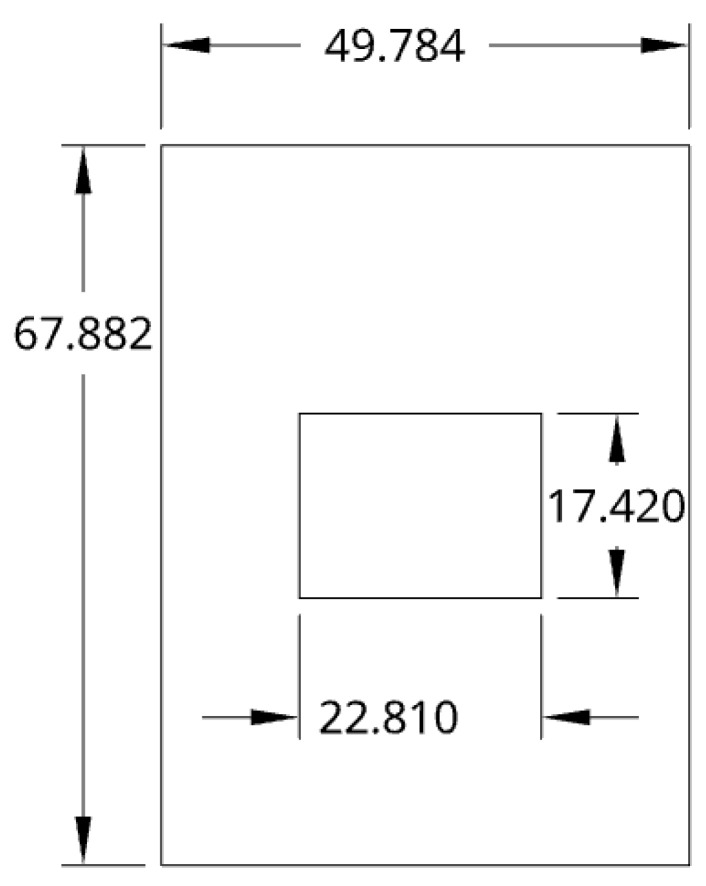
Dimensions of the patch antenna/sensor in mm.

Patch antenna design plays a critical role in sensing performance by determining the resonant frequency of a patch antenna. Rectangular patches were chosen over similar dipole and circular patches for their narrow bandwidth, which is advantageous for antennas operating at a single resonant frequency [1,45]. The resonant frequency of the sensor is influenced by physical parameters such as the substrate material, substrate height, and patch dimensions. Heating or cooling the patch antenna changes the dielectric constant of the substrate, altering the resonant frequency [29]. The relation between resonant frequency and dielectric constant is given by

(1) $$\begin{array}{c} f_{0}=\frac{c}{2L\sqrt{\varepsilon_{r}}} \end{array}$$

where *c* is the speed of light, *L* is the length of the conductive patch, $f_{0}$ is the $TM_{10}$ resonant frequency of the patch antenna, and $\varepsilon_{r}$ is the dielectric constant [29]. The $TM_{10}$ mode is the fundamental resonance, with the electric field varying along the length of the patch, while the $TM_{01}$ mode varies along the width. Higher-order harmonics (e.g., $TM_{20}$, $TM_{30}$) exhibit significantly weaker resonance absorption and lower amplitude due to reduced coupling efficiency and radiation strength at those frequencies. These harmonics fall outside the optimal operational range of the UWB antennas used in this study, which were designed to maximize sensitivity around the fundamental resonance. The fundamental mode remains the most reliable and informative for temperature sensing in this system. The transmission line model was used to calculate the dimensions of the radiating patch in order to create a sensor with a desired resonant frequency. The length (*L*) and width (*W*) of the antenna’s patch depended on the desired resonant frequency ($f_{0}$) and the dielectric constant ($\varepsilon_{r}$) of the substrate, which was calculated as

(2) $$W=\frac{c}{2f_{0}\sqrt{\frac{\varepsilon_{r}+1}{2}}}$$

(3) $$\varepsilon_{\mathrm{eff}}=\frac{\varepsilon_{r}+1}{2}+\frac{\varepsilon_{r}-1}{2}{\left[1+12\left(\frac{h}{W}\right)\right]}^{-1/2}$$

(4) $$L=\frac{c}{2f_{0}\sqrt{\varepsilon_{\mathrm{eff}}}}-0.824h\left(\frac{\left(\varepsilon_{\mathrm{eff}}+0.3\right)\left(\frac{W}{h}+0.264\right)}{\left(\varepsilon_{\mathrm{eff}}-0.258\right)\left(\frac{W}{h}+0.8\right)}\right)$$

where $\varepsilon_{\mathrm{eff}}$ is the effective dielectric constant due to the fringing effect, and *h* is the height of the substrate. The resonant frequency of the patch was designed to be 4.0 GHz to balance antenna size and compatibility with the frequency range of the interrogation antennas. The substrate between the ground plane and the top conducting patch was FR4 with a dielectric constant of 4.4, a temperature coefficient of the dielectric constant (TCD) of −200 ppm/°C [46], and a substrate height of 1.53 mm. The resulting dimensions of the patch using Equations (2) and (4) were a width of 22.81 mm and a length of 17.42 mm. Figure 1 shows the design using the solved *L* and *W*. Copper was selected for the material of the patch for its conductivity of $\sigma_{\mathrm{copper}}=5.96\times 10^{7}\;S/m$ and widespread availability in standard PCB fabrication processes [47]. The copper patch was directed outwards due to the radiation pattern of the patch antenna, facing the interrogator antennas, and the E-Planes of both the patch antenna and interrogator antennas were coplanar. Copper is not suitable for environments exceeding 1000 °C, such as those found in industrial boiler systems, which can reach temperatures up to 1500 °C [1]. The focus of this work was to develop and validate a machine learning–based calibration pipeline with available materials. This methodology can be extended to high-temperature materials, as demonstrated in prior work [1,7]. Transitioning to high-temperature-capable materials will involve both material and fabrication considerations. Copper and FR4 could be replaced with materials such as indium tin oxide (ITO), silver, or steel for the patch, and ceramic substrates like alumina or LTCC, which offer stable dielectric properties and mechanical robustness at harsh temperatures. This transition will require the adoption of specialized deposition techniques such as screen printing or inkjet printing of conductive oxides onto sintered ceramic substrates. Co-firing or thermal post-processing steps may be necessary to ensure adhesion and electrical performance. The feasibility of this transition is supported by recent ceramic sensor work [1,7], and the machine learning framework developed here remains applicable, as these high-temperature substrates also exhibit temperature-dependent resonance shifts that can be learned and calibrated using ML models.

**Figure 2:**
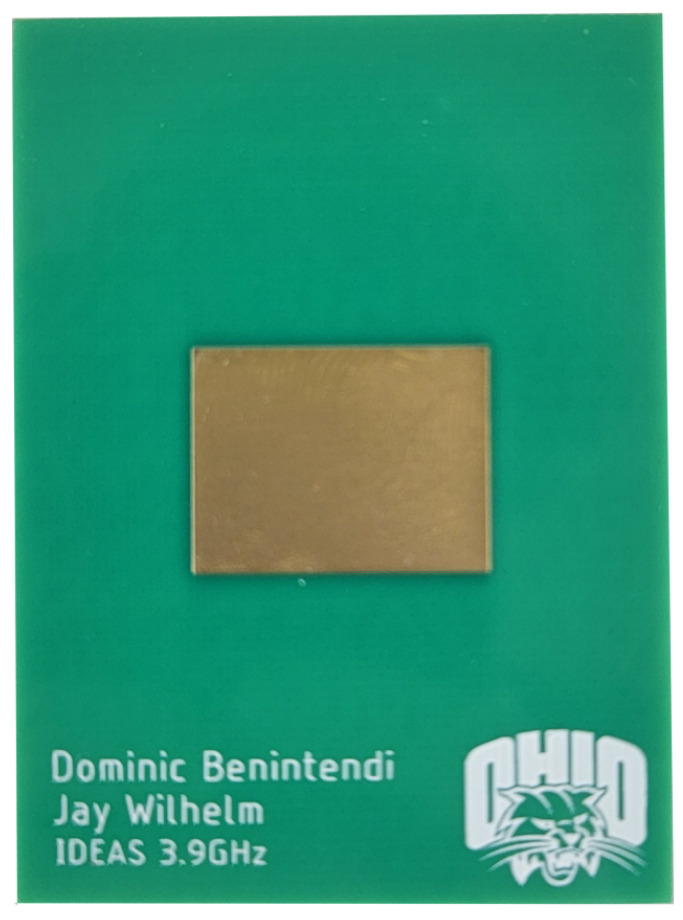
Final iteration of the sensor.

**Figure 3:**
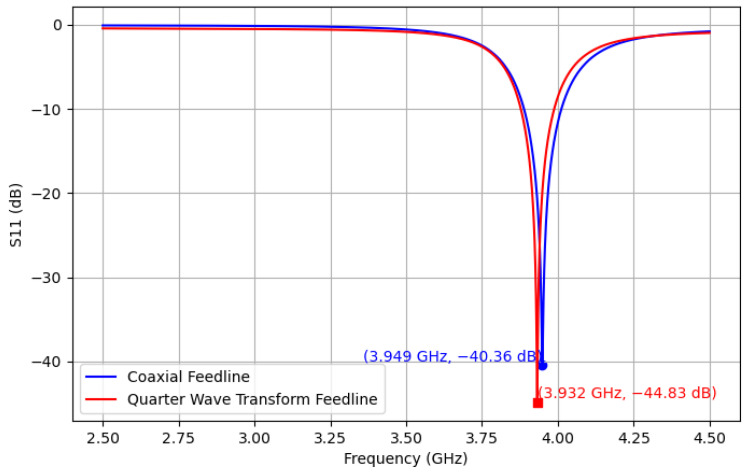
Coaxial and quarter-wave feedline patch antenna $S_{11}$ curves.

**Figure 4:**
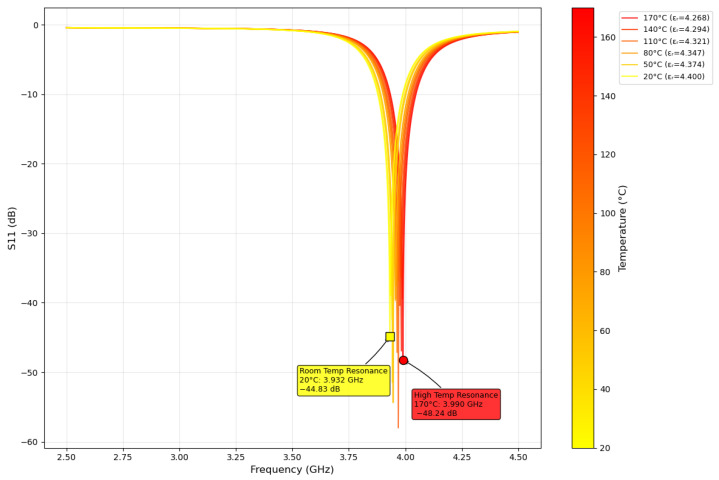
Quarter-wave patch antenna vs. temperature; dielectric constant change was simulated using the TCD of the substrate.

HFSS ANSYS (2024 R2) [48] simulations were used to verify the estimated theoretical resonant frequency of 4.0 GHz. Two feedline configurations were modeled in HFSS ANSYS: (1) quarter-wave transformer and (2) coaxial. The final design iteration of the patch sensor used in this work is shown in Figure 2. Antenna characterization results showed a resonant frequency of 3.949 GHz from the coaxial feedline and 3.932 GHz from the quarter-wave transformer, as shown in Figure 3. Radiation simulations showed a dBi of 4.18 at the main lobe of the system. The quarter-wave patch antenna’s changes under temperature from 20 °C to 170 °C were simulated by parametrically changing the dielectric constant using the TCD of the substrate, as shown in Figure 4. Ensuring a 50 Ω impedance match was essential for accurately estimating the TM_10_ resonant frequency along the patch length.

### 2.2. Experimental Setup

**Figure 5:**
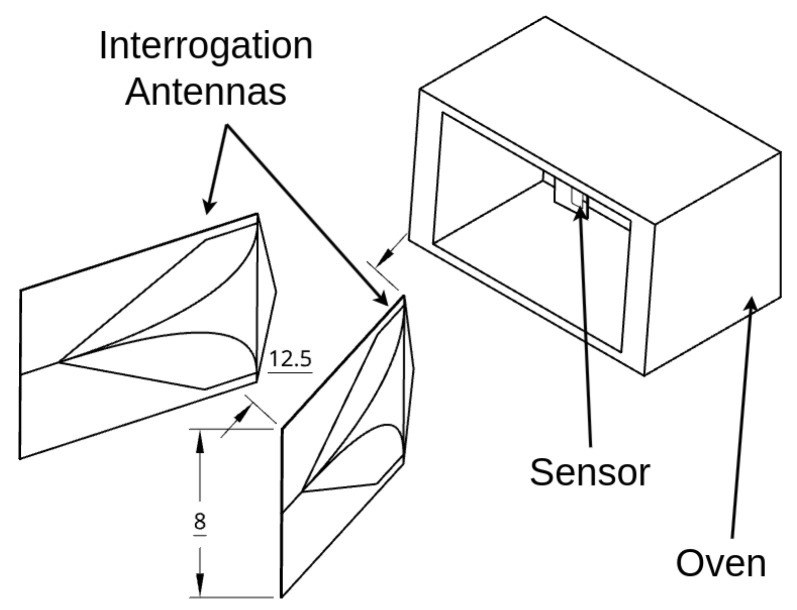
Experimental setup including the two interrogator antennas, oven, and sensor.

**Figure 6:**
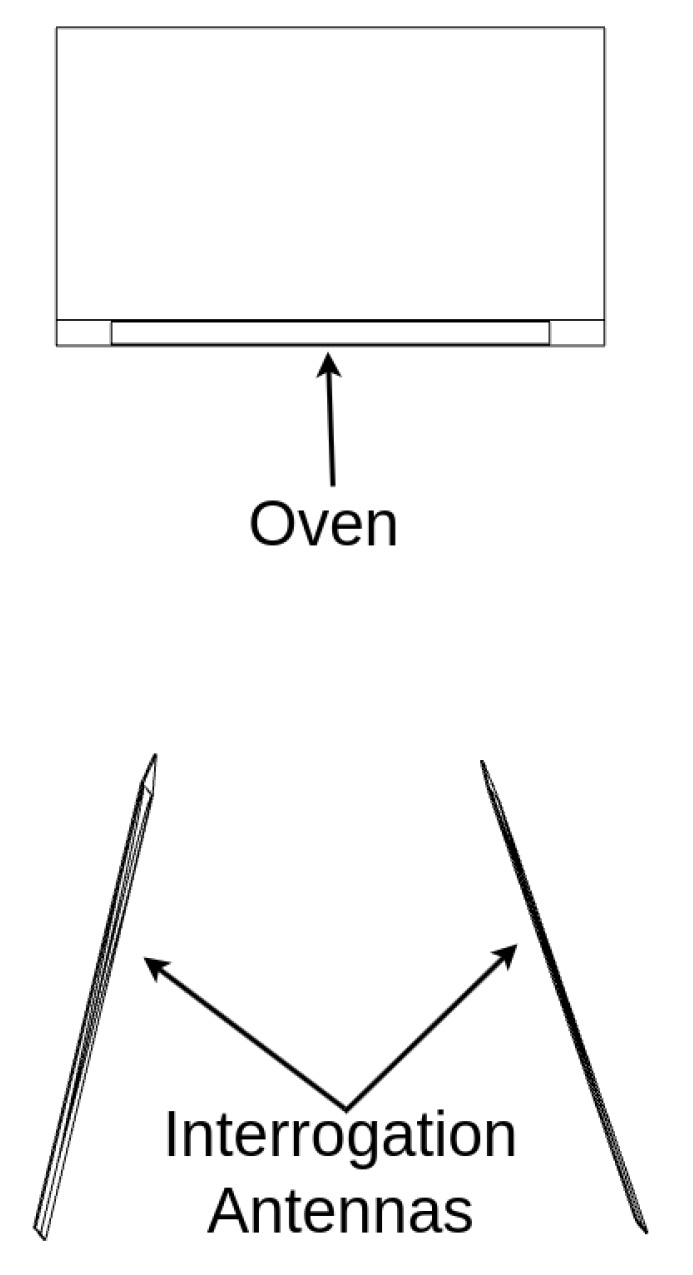
Experimental setup from a top–down view.

Wireless interrogation and live monitoring of a patch sensor’s resonance behavior in a heated environment were required to characterize the sensor’s response to temperature changes. The experimental setup included an oven, a patch antenna, and two interrogation antennas to monitor shifts in the patch’s resonance response as the oven temperature changed, as shown in Figure 5 and Figure 6. The VNA recorded the resonance response, which was processed by a data historian, while a thermocouple simultaneously recorded temperature data for use in training datasets. Vector network analysis using the LibreVNA [49] captured forward transmission measurements represented by $S_{21}$ data, indicating the signals received at port 2 from port 1. These measurements were collected using a pair of high-gain Ultra Wideband (UWB) antennas. The patch antenna was placed in an oven to simulate operating temperatures between 20 °C and 170 °C, enabling thermal calibration over the oven’s expected range of use. Time-gating was applied to each recorded signal to isolate reflections and suppress interference from environmental multipath, improving signal clarity.

Received $S_{21}$ signals were collected under varying temperatures by heating the patch antenna from 20 °C to 170 °C using a Hamilton Beach Model 31403 oven. The patch sensor was placed in the middle of the oven to maintain a direct line of sight to the interrogation antennas. The true temperature of the sensor was recorded using a T-type thermocouple connected to a Measurement Computing USB 2001-TC device. The end of the thermocouple was affixed to the ground plane of the sensor’s back, away from the line of interrogation, using Kapton tape, for a heat resistance of 260 °C [50]. The oven utilized a Proportional–Integral–Derivative (PID) controller for direct temperature control over a programmed time period.

The interrogator antennas used were a pair of RFSPACE TSA600 Ultra-Wide Band log-periodic antennas with 12 dBi gain at 4 GHz. The interrogator antennas were positioned adjacent to the oven opening at variable distances from a sensor and were connected to the VNA using 0.3 m coaxial cables. Both the interrogator antennas and the patch sensor were linearly polarized with the electric field oriented vertically (co-polarized), and the port plane was defined at the antenna–cable junction. This polarization and orientation setup was maintained consistently across all test configurations. Short-Open-Load-Through (SOLT) calibration ensured measurement accuracy.

Reflected signal data was recorded as the $S_{21}$ parameter using the LibreVNA [49], an affordable open-source 2-port Vector Network Analyzer covering 100 kHz to 6 GHz. The data recorded by the VNA was transmitted to a data historian using Standard Commands for Programmable Instruments (SCPI), a standard protocol for sending and receiving device data over Internet Protocol (IP) provided with the LibreVNA. The Intermediate Frequency Bandwidth (IFBW) was set to 1 kHz to mitigate noise and improve measurement accuracy. The transmitting antenna emitted at a power level of 2 dBm, the maximum supported by the VNA, while each $S_{21}$ signal was sampled with 4000 points across a 1 GHz frequency range. The 1 GHz span was selected to preserve fine time-domain resolution (approximately 1 ns) necessary for effective time-gating and isolation of the antenna’s primary reflection response. A high point count of 4000 was chosen to ensure sufficient frequency-domain resolution for accurately identifying resonance features. However, this combination of wide bandwidth and dense sampling increases the VNA sweep time, resulting in a polling time of approximately 10 s per measurement, which is a trade-off optimized for balancing temporal resolution, frequency precision, and data collection speed.

### 2.3. Time Gating

**Figure 7:**
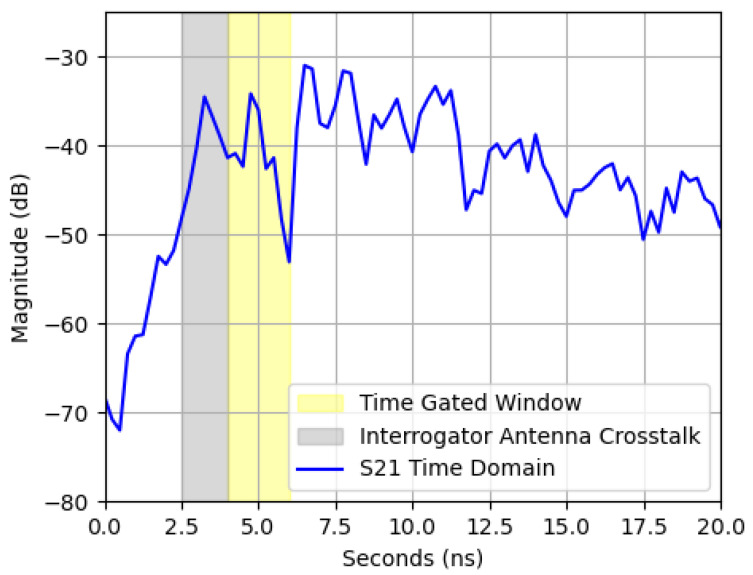
Example of time-gating using a dataset recorded with a distance of 350 mm.

Accurate characterization of the patch antenna’s response required isolating its signal from environmental reflections and interrogator antenna crosstalk. Multipath interference and background reflections in ovens with complex geometries obscured the true resonance behavior of the sensor. Time-domain processing offered a means to distinguish between reflections based on their propagation delays, allowing for selective extraction of a sensor’s backscattered signal. The interrogator antenna at the VNA port 1 transmitted a signal toward the patch antenna, which reflected it back toward the antenna at the VNA port 2. Signals at the patch antenna’s resonant frequency were absorbed and re-emitted over a delay of approximately 100 ns, preventing them from reaching the VNA port 2 within the 8 ns time window typically associated with direct reflections. Reflections from background objects introduced additional noise into the final $S_{21}$ measurement. Time-gating mitigated these unwanted reflections, isolating the patch antenna’s response and reducing noise [4,8,11,12], as shown in Figure 7. The first reflection on the $S_{21}$ time-domain signal resulted from crosstalk between the interrogator antennas. The second came from backscattering off the patch antenna, and subsequent reflections originated from the oven interior and surroundings. Gaussian windowing with a standard deviation of 0.4 was applied to the signal to isolate the desired portion.

The VNA $S_{21}$ signal was converted into the time domain using an Inverse Fast Fourier Transform (IFFT) algorithm. The period during which the interrogator antenna received reflections from the patch antenna was isolated using Gaussian windowing, which was applied to the time-domain signal. The Gaussian windowing function tapered the signal, allowing for smooth transitions at the edges of the window and minimizing spectral leakage during the FFT. The signal re-entered the frequency domain using a Fast Fourier Transform (FFT) after applying the window. The resulting $TGS_{21}$ signal more accurately represented the frequency response of the patch antenna and mitigated unknown variables in the environment, such as antenna crosstalk. The Gaussian window range was determined by estimating the arrival time for the antenna’s backscattered signal using the equation

(5) $$\begin{array}{cc} T_{\mathrm{offset}} & =\frac{D_{\mathrm{total}}}{c}\\ \; & =\frac{2\;\cdot \;(L_{\mathrm{antenna}}+D_{\mathrm{sensor}})}{c} \end{array}$$

where $L_{\mathrm{antenna}}$ was the effective length of the interrogator antennas, and $D_{\mathrm{sensor}}$ was the distance from the tip of one interrogator antenna to the sensor. Time-gating ranges were created using Equation (5) based on different known values of $D_{sensor}$, as shown in Table 1.

The patch antenna resonant frequency was isolated within the time-gated $S_{21}$ signal during live testing, a process known as resonant point isolation. Viewing the signal in LibreVNA and slightly adjusting antenna positions revealed a clear resonant point. IFBW was increased to 50 kHz to improve the refresh rate, enabling quick probing of a sensor’s resonant point while sacrificing accuracy. Once the resonant point was isolated, the IFBW was restored to 1 kHz, the oven was turned on, and data collection began.

### 2.4. Data Processing System

**Figure 8:**
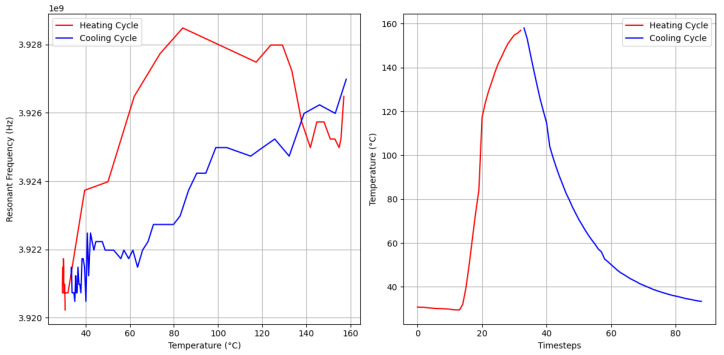
Hysteresis of the sensor: The sensor underwent rapid temperature change, showcasing the hysteresis effect. Plot on the left shows how resonant frequency changes with time. Plot on the right shows the rapid temperature changes.

Hysteresis, where a system’s behavior depends on its history, was observed as the resonant frequency shifts lagged when a sensor was rapidly heated or cooled, as shown in Figure 8. Hysteresis was experienced and discussed in Idhaiam’s work but was mitigated by reducing the heating rate [7]. Birdsell et al. mitigated hysteresis by waiting 30 min after reaching the target temperature to allow the sensor to stabilize [6]. Boiler sensor applications cannot control the heating rate, requiring hysteresis to be addressed without altering heating conditions, which necessitated a temporally aware model. LSTM models may recognize whether a sensor is heating up or cooling down using the past *N* timesteps, offering more accurate estimations than a linear regression algorithm. The LSTM model can learn the directional behavior of the sensor response by leveraging temporal patterns in the input sequence, effectively compensating for hysteresis without modifying the thermal environment.

The data historian was a processing step that utilized a custom-made Python package (v1.0) that facilitated analysis and cataloging of data from the VNA and created trainable datasets for the machine learning models. Time-gated $S_{21}$ signals were sent to the data historian via the SCPI protocol from the LibreVNA. Live views of temperature estimation, resonance response versus temperature, $S_{21}$ waterfall plots, frequency sweeps, and resonant frequency over time were provided through the live graphical user interface. The polling time between each time step varied depending on factors such as IFBW, the range of the frequency sweep, and the number of points. Each dataset required approximately 10 s for each time step with the previously provided VNA parameters. The overall polling time depended on the slowest polling rate between the VNA and thermocouple, as both devices operated concurrently. The slowest polling time was imposed by the VNA.

**Figure 9:**
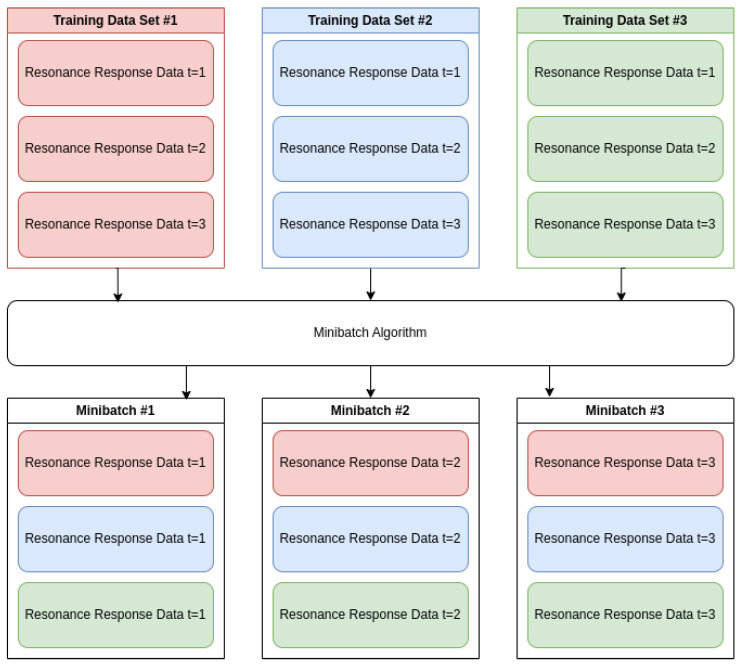
Shows the algorithm behind minibatching multiple datasets.

Raw $S_{21}$ data collected from the LibreVNA lacked structure and required processing to extract meaningful features for model training. Unstructured $S_{21}$ data was processed and organized using the data historian, which allowed for feature extraction, live data analysis, and model training. Inconsistent input ranges in the raw feature data hindered model convergence and prediction stability. Data preprocessing was used to prepare the datasets for machine learning models and refine the raw data collected from the LibreVNA. Preprocessing steps included minibatching, timestepping, and scaling. Minibatching shuffled chunks from multiple datasets, as shown in Figure 9, which prevented overfitting on the final dataset within an epoch and improved model generalization across all datasets. Timesteps were implemented for the LSTM by including 15 previous points per sample, with earlier data points in a dataset (N < 15) being truncated. Normalization was required to ensure that all input features contributed proportionally during training and inference, preventing features with larger numerical ranges from dominating the learning process. Features with bounded, monotonic behavior were normalized using MinMax scaling to ensure consistent input ranges between 0 and 1 for model training, as summarized in Table 2. MinMax scaling was preferred over standard scaling because the features exhibited linear trends rather than mean-centered distributions, making MinMax scaling more effective at preserving trend information critical to model performance.

### 2.5. Training a Model

Machine learning models were trained to estimate temperature from resonance data collected during experimental trials. Seventy percent of the 28 preprocessed datasets were used for training, and the remaining thirty percent were reserved for validation. The experimental setup provided sufficient variation in temperature and interrogation distance to support effective model learning. Several models were trained and evaluated, including linear regression, a fully connected neural network, and a Long Short-Term Memory (LSTM) model, to identify the best-performing estimator for sensor calibration. Models were then compared using MAE and $R^{2}$ validation metrics. Model weights were saved for runtime inference once validated, allowing the system to generate live temperature estimates from real-time $S_{21}$ input. The training-validation dataset pair enabled the ML-based sensor calibration for live temperature estimation.

**Figure 10:**
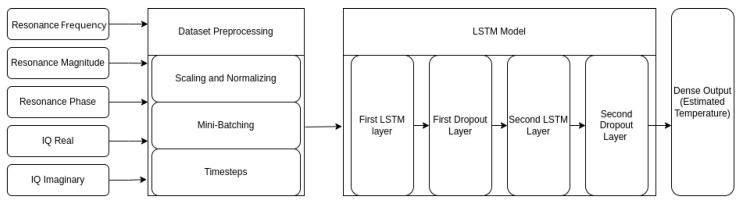
LSTM model overview. Contains two LSTM Layers and two dropout layers to prevent overfitting.

The LSTM and NN used the TensorFlow library to integrate with the rest of the data processing pipeline. Hyper-parameters, which were variables that described the overall structure of the model, were adjusted heuristically using Bayesian optimization to enhance the model’s performance and minimize the Mean Absolute Error (MAE). Some examples of these hyper-parameters include the LSTM model sizes (neuron percentage, neuron shrink), learning rate, kernel regularizer, and dropout layer percentage, as shown in Table 3. The final model architecture consisted of six layers. The first layer was an input layer with a shape of timesteps×features. The number of timesteps for these tests was 15. The features fed into the models were normalized resonant frequency, resonant magnitude, resonant phase, and IQ data (imaginary and real). The first LSTM layer had 213 neurons, the second had 49 neurons, and each layer used the tanh activation function to promote profiling of non-linear functions. The l2 kernel regularizer of the model was set to 0.00094. Each LSTM layer was paired with a dropout layer, as shown in Figure 10. Dropout layers randomly deactivate connections within the model to reduce overfitting on the data. Each dropout layer was set to drop 0.018% of neurons during each forward pass. MAE was chosen as the loss function due to its suitability for regression tasks. MAE calculated the absolute differences between predicted values and their actual outcomes. Linear regression on the normalized resonant frequency provided a baseline for comparison. NN used the same input features and hyperparameters as the LSTM model, excluding timesteps. The absence of temporal information prevented NN from capturing patterns across time.

**Figure 11:**
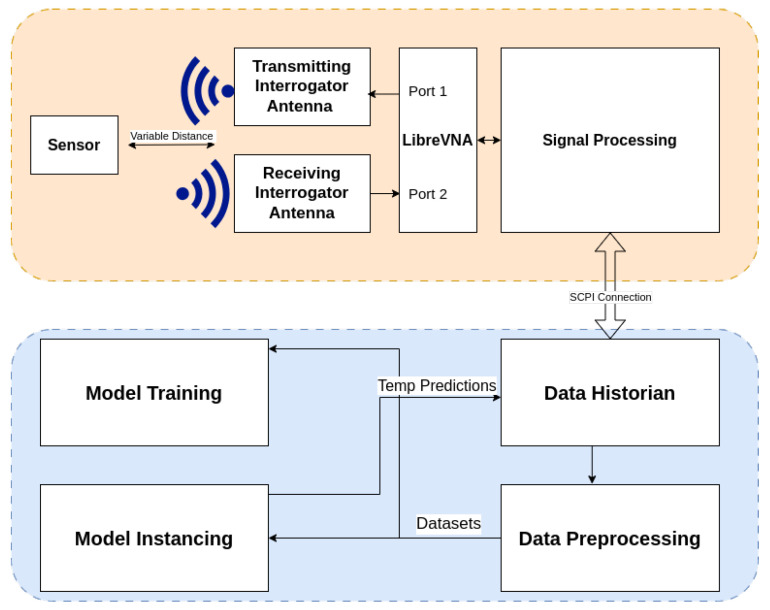
Overview of the system architecture, including sensor interrogation (orange) and dataset processing (blue) components.

The complete interrogation and model training pipeline is shown in Figure 11, with sensor interrogation in orange and dataset processing in blue. The top section depicts the physical interrogation setup, where a passive wireless sensor is positioned at a variable distance from a pair of interrogator antennas. The transmitting antenna connects to port 1 of the VNA, while the receiving antenna connects to port 2. The system interrogates the sensor wirelessly by sweeping a range of frequencies and measuring the transmitted $S_{21}$ signal response, which is processed using time-gating. Signal features are forwarded to the data historian using a Standard Commands for Programmable Instruments (SCPI) connection. The historian stores the incoming data and forwards it to a preprocessing stage, which cleans and formats the signal for ML workflows. Processed datasets are used to train models, which generate temperature predictions from incoming data. Predictions are looped back into the historian for further validation and monitoring.

## 3. Results and Discussion

The automated calibration system relied on data from 28 experimental datasets of resonance measurements. Each dataset captured the patch antenna’s resonance response under varying thermal conditions and interrogation distances to evaluate the impact of these conditions on sensitivity and accuracy while also providing the model with diverse training scenarios. The datasets were collected under both rising and falling temperature conditions across five interrogation distances: 200 mm, 350 mm, 500 mm, 750 mm, and 900 mm. At 200 mm, three rising and four falling temperature datasets were recorded. At 350 mm, four rising and three falling datasets were collected. At 500 mm, four rising and four falling datasets were recorded. At 750 mm, two rising and two falling datasets were recorded. At 900 mm, one rising and one falling dataset were recorded. Most datasets were collected using the original patch antenna sensor from previous work [1], while four datasets were recorded using the sensor fabricated during this study, with no difference observed between the sensors’ performance. The distribution of datasets ensured that both thermal directions were represented across all distances used in the study, which is shown in Table 4. The data indicated measurable degradation at extreme ranges beyond 750 mm for the setup utilized. Multiple ML models were evaluated, and the LSTM architecture demonstrated the highest accuracy and generalization between validation datasets.

**Figure 12:**
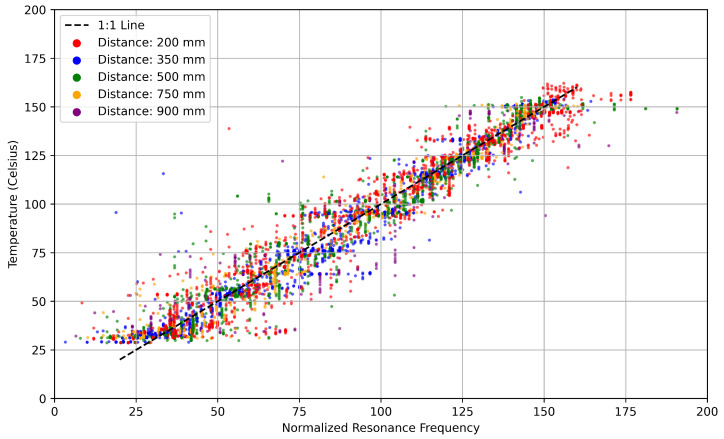
Normalized resonant frequency vs. temperature across 28 datasets, with each color representing a distance group.

Testing of the patch antennas’ resonance behavior revealed that the initial resonant frequency of the interrogated $S_{21}$ signals was approximately 3.85 GHz, with values varying between datasets, ranging from 3.60 GHz to 3.95 GHz. Similar variations were observed by Tennant et al., where the initial resonant frequency of each test ranged from 3.0 GHz to 3.1 GHz [1]. This work found that variations in the initial resonant frequency of the patch antennas between datasets resulted from the time-gating algorithm. Slight variations in the distances between the interrogator antennas and a sensor, along with the limited time-domain resolution, introduced small deviations in a sensor’s apparent initial resonant frequency. Time-gating remained essential for eliminating crosstalk and background noise despite the inconsistent initial resonant frequency. Normalized changes in resonant frequency were provided to the model rather than absolute resonant frequencies to address the varying starting resonant frequency, as demonstrated in Yao’s work [4]. The normalized resonant frequency was determined by solving for $\Delta f_{\mathrm{rel}}$

(6) $$\Delta f_{\mathrm{rel}}=\frac{f-f_{0}}{m}$$

where *f* was the resonant frequency at any given temperature, $f_{0}$ was the resonant frequency at 0 °C and *m* was the slope of the frequency change per degree Celsius, also known as the sensitivity, which is computed per dataset. The normalized frequencies versus temperatures for all 28 datasets are shown in Figure 12. The system required configuration before live instancing by determining the resonant frequency at two known temperatures due to the use of the normalized resonant frequency.

**Figure 13:**
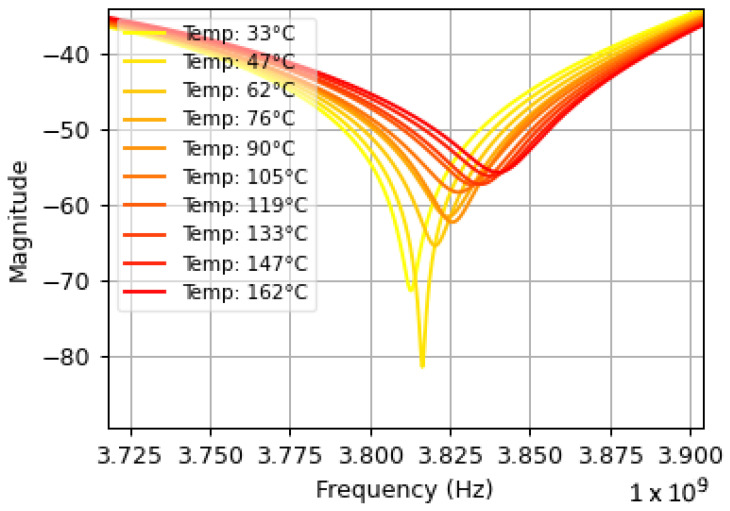
Resonance response vs. temperature at 200 mm. Resonant frequency shifts to the right as temperature increases and magnitude decreases.

**Figure 14:**
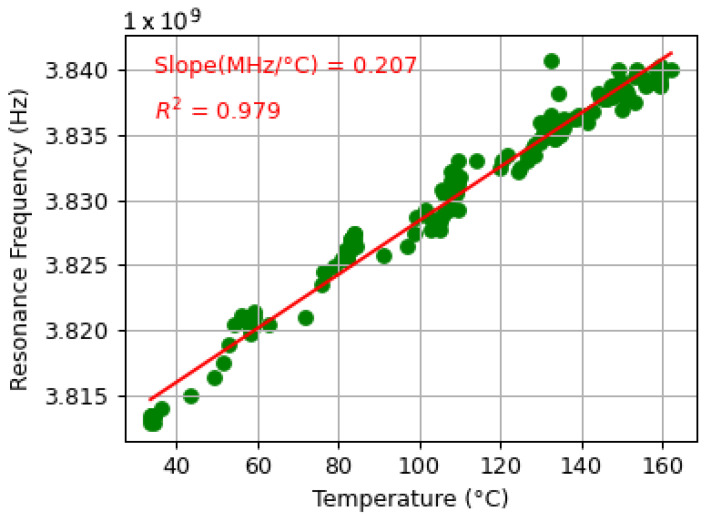
Time-gated resonant frequency vs. temperature at 200 mm. The dataset shows a linear correlation between resonant frequency and temperature (20–160 °C).

Each dataset consisted of a sensor’s resonance response as it was subjected to temperatures varying between 20 °C and 170 °C. Some datasets captured sensor cooling, while others captured sensor heating. Variations in temperature acceleration, sudden jumps in temperature, and slow periods of heating and cooling were included to provide the model with a more general understanding of a real-world boiler system. Each dataset demonstrated a linear correlation between the resonant frequency of the patch antenna and the temperature recorded by the thermocouple. Sample data interrogated from the original patch antenna sensor [1] is shown in Figure 13 and Figure 14, where Figure 13 illustrates the $TGS_{21}$ readings at various temperatures, and Figure 14 shows how the resonant frequency derived from those readings correlates with temperature. The slope of the line of best fit shown in Figure 14 represents the sensitivity of the sensor. The sensitivities between datasets varied depending on distance and the time-gating window.

**Figure 15:**
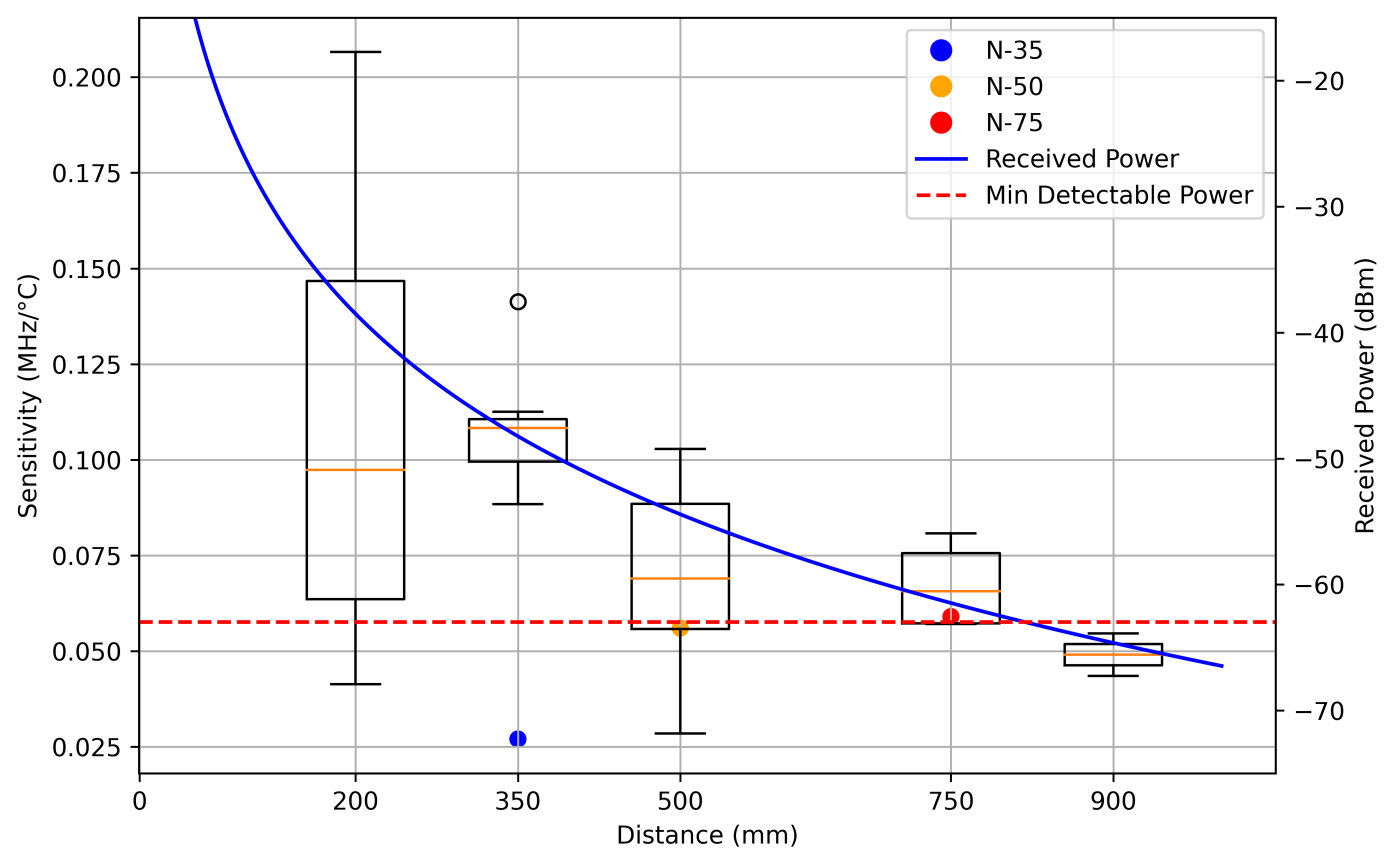
Sensor sensitivities vs. distance. Received power is theoretical estimation of the received power at the receiving interrogation antenna using Friis transmission equation.

Sensor sensitivity exponentially decreased as distance from the antennas increased, as shown in Figure 15, with sensitivities approaching 0.2 $\frac{MHz}{{}^{\circ}C}$ at 200 mm and declining to 0.05 $\frac{MHz}{{}^{\circ}C}$ at 900 mm. The theoretical maximum sensitivity given a temperature coefficient of dielectric constant (TCD) of −200 ppm/°C would be approximately 0.4 $\frac{MHz}{{}^{\circ}C}$. Theoretical analysis of the maximum interrogation distance for a wireless patch antenna system was completed using the Friis transmission equation [4]. The received power $P_{r}$ at the receiving interrogation antenna is modeled by,

(7) $$P_{r}=P_{t}\;\cdot \;G_{t}\;\cdot \;G_{r}\;\cdot \;G_{s}^{2}\;\cdot \;\left|S_{11}\right|\;\cdot \;L_{d}\;\cdot \;{\left(\frac{\lambda }{4\pi d}\right)}^{4}$$

where $P_{t}$ is the transmitted power, $G_{t}$ and $G_{r}$ are the transmit and receive antenna gains, $G_{s}$ is the gain of the patch sensor, $|S_{11}|$ is the magnitude of the reflection coefficient at resonance, $L_{d}$ accounts for insertion loss, λ is the wavelength, and *d* is the interrogation distance. The minimum detectable power $P_{\min}$, below which signal detection is unreliable, is calculated using the thermal noise equation,

(8) $$P_{\min}=k_{B}TBF\;\cdot \;\mathrm{SNR}$$

where $k_{B}$ is Boltzmann’s constant, *T* is the system temperature at room temperature, *B* is the bandwidth, *F* is the noise figure of the receiver, and SNR is the required signal-to-noise ratio. The maximum interrogation range $d_{\max}$ is estimated by solving for distance *d* when the received power $P_{r}$ equals the minimum detectable power $P_{\min}$:

(9) $$d_{\max}=\frac{\lambda }{4\pi }{\left(\frac{P_{t}G_{t}G_{r}G_{s}^{2}\left|S_{11}\right|L_{d}}{k_{B}TBF\;\cdot \;\mathrm{SNR}}\right)}^{\frac{1}{4}}$$

**Figure 16:**
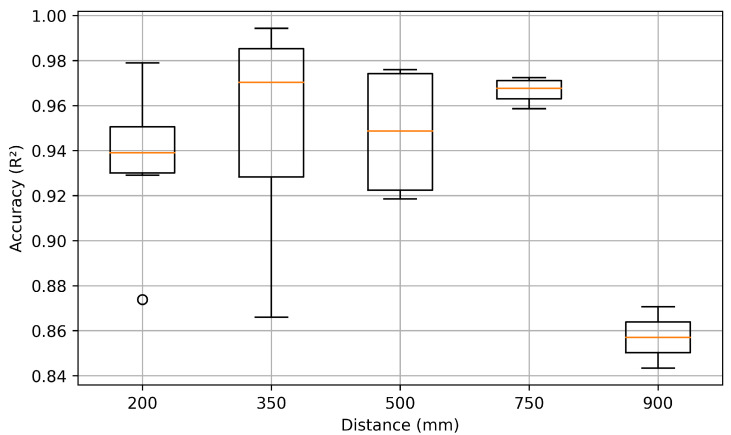
$R^{2}$ Score vs. Distance.

Parameter values such as a 4.0 GHz resonance frequency, 12 dBi interrogator antenna gain, 4.2 dBi sensor gain, and representative estimates for path loss, noise figure, and thermal noise were used in this calculation. The analysis predicted a maximum effective interrogation distance of approximately 800 mm, which aligned with experimental observations shown in Figure 15 and Figure 16. The high variation in sensitivity for datasets at 200 mm was due to difficulty maintaining time-gating isolation between crosstalk and antenna reflections at close range. Findings reported by Tennant et al. indicated by the three points labeled N-35, N-50, and N-75 in Figure 15, contradicted this work as they observed that sensitivity increased with distance [1]. Tennant et al. observed sensitivity values ranging from 22 to 62 $\frac{KHz}{{}^{\circ}C}$, with sensitivity increasing as the interrogation distance increased from 350 mm to 750 mm [1]. Low sensitivity observed at a 350 mm measurement in their study may have resulted from the increased difficulty of time-gating at close ranges or from the decreased conductivity of the indium tin oxide (ITO) patch material used. Ma et al. found that sensitivity exponentially decays with distance [13], which aligns with the results found in this work. Cheng et al. showed sensitivity values of 0.41–0.58 $\frac{MHz}{{}^{\circ}C}$ for their 5.0 GHz patch antenna interrogated at a distance of 30 mm, though the distance between the interrogation antenna and the sensor was fixed [11]. The loss associated with low sensitivities could be mitigated by using higher-resolution $S_{21}$ readings, although this approach would inversely impact polling times.

Accuracy varied significantly with interrogation distance. Broader antenna reception cones captured excessive background noise at 900 mm, pushing the $S_{21}$ signal closer to the noise floor and resulting in a noticeably lower $R^{2}$ score, as shown in Figure 16. The 900 mm datasets were excluded from training due to the significant degradation in received signal quality at that range, which resulted in noisy and unreliable measurements. Including these data points would have introduced distortions that impair model performance within the effective operating range of 200–750 mm. Measurements at 200 mm also exhibited low $R^{2}$ scores, largely due to difficulties in applying a precise time-gating window at such close range. The results highlighted the importance of optimal interrogation distance for accurate temperature estimation.

**Figure 17:**
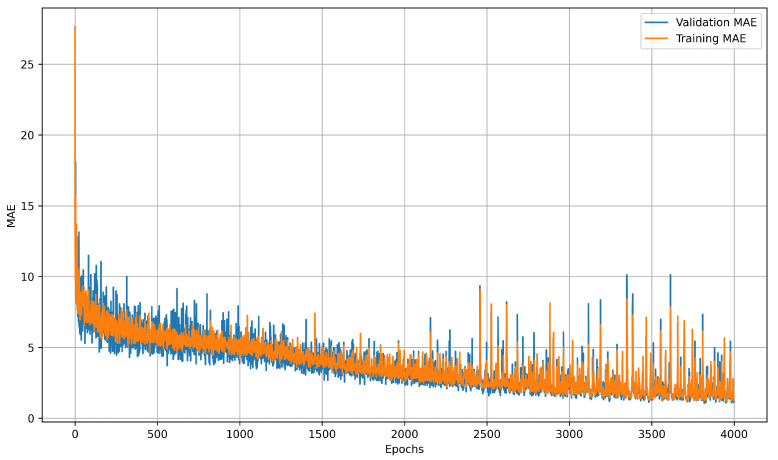
LSTM model learning curves after 4000 epochs. Learning curves were recorded using MAE as the loss function.

**Figure 18:**
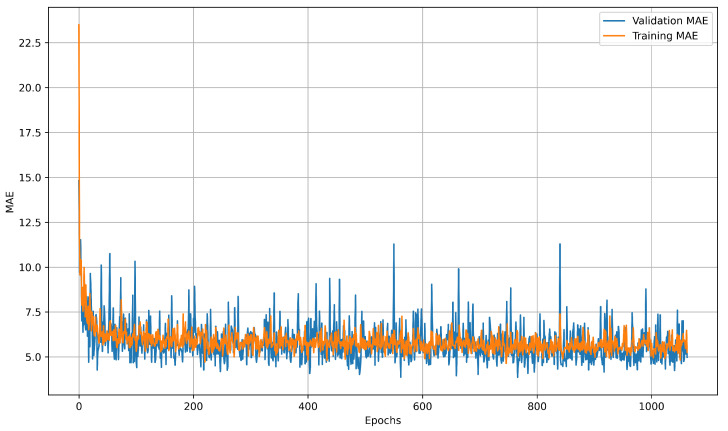
NN Learning curves after 4000 epochs. Early stopping was triggered at epoch 1026. Learning curves were recorded using MAE as the loss function.

Learning curves illustrate how prediction error changes throughout training, offering insight into model convergence, generalization, and potential issues such as underfitting or overfitting. The training curve reflects performance on known data, while the validation curve measures accuracy on unseen data, with divergence between them often indicating overfitting. LSTM learning curves showed diminishing returns after 4000 epochs on the preprocessed collection of 28 datasets. MAE stagnated around 1.32 °C after the 3500-epoch threshold, as shown in Figure 17. The NN model plateaued much earlier, reaching minimal improvement beyond 200 epochs, as illustrated in Figure 18. These learning curves highlight the LSTM model’s capacity for continued refinement over extended training periods, while the NN model rapidly converged to its optimal performance.

**Figure 19:**
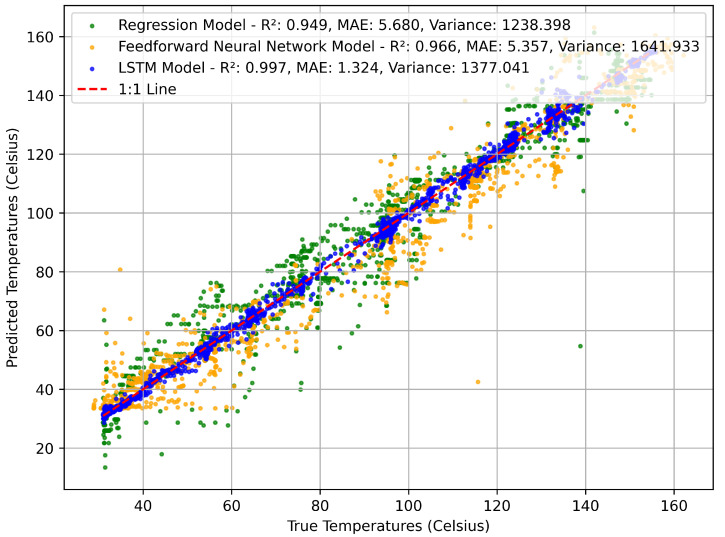
Comparison of prediction accuracy between the three types of models on a validation dataset.

Each trained model was evaluated on a validation dataset to assess generalization performance. Model validation tested the models’ ability to make accurate temperature predictions on previously unseen data, providing a fair comparison of model architectures under realistic deployment conditions. LSTM outperformed all other tested model architectures in both MAE and $R^{2}$. Each model’s performance when predicting the temperature is shown in Figure 19, where models with data points closer to the line of best fit provided more accurate estimates. The LSTM performed with an $R^{2}$ score of 0.997, while the traditional NN and LR models showed $R^{2}$ scores of 0.966 and 0.949 respectively. Higher variance in LSTM predictions does not imply inaccuracy, as the model still achieved superior correlation and lower average error. Temperature predictions were off by an average of 1.32 °C for the LSTM model, compared with 5.36 °C for the NN and 5.68 °C for linear regression. The LSTM model improved over linear regression by 76% in terms of MAE. The model demonstrates reliable performance within a temperature range of 20–170 °C, interrogation distances between 200 and 750 mm, and under heating rates below approximately 1.5 °C/s; conditions outside these bounds may lead to increased prediction error. The summarized results of each model are shown in Table 5. Comparisons between existing sensing technologies and the model are provided in Table 6.

## 4. Conclusions

Calibration of wireless patch antennas for temperature monitoring in extreme environments using machine learning was investigated. The overall goal was to use machine learning to automate calibration for improved sensor accuracy. Enabling our novel automated sensor calibration system was achieved by combining time-gated resonance response features with an LSTM model during temperature and frequency scans. The system was calibrated for hysteresis using an ML-driven methodology rather than traditional linear regression, and it was specifically developed for live operation with polling intervals as low as 10 s. The distance between the patch sensor and the interrogator antenna identified an exponential inverse relationship matching RF theory.

Previous work required the use of post-processing and manual calibration, whereas our setup and method functioned in real time with automated calibration. Data collected from the developed process demonstrated sensitivities of 0.052–0.20 $\frac{MHz}{{}^{\circ}C}$ with a 76% improvement in error when using an LSTM-based calibration method. The experiments highlighted an innovative solution for patch antenna-based non-contact temperature measurement, which addresses several limitations with conventional methods such as RFID-based systems, infrared, and thermocouples. Wireless characterization of patch antennas using machine learning enabled an alternative in situ temperature measurement system, which will impact predictive maintenance and industrial monitoring with broader applicability. Future work may explore amplifier integration to increase the effective range and signal clarity, potentially enabling accurate measurements beyond 750 mm.

## Figures and Tables

**Table 1:** Round-trip time for patch antenna backscattering in the $S_{21}$ signal and the corresponding time-gating range.

Distance from Antenna to Sensor (mm)	Estimated Travel Time (ns)	Windowing Range (ns)
200	3.43	3–7
350	4.43	4–8
500	5.43	6–9
750	7.10	7–11
900	8.11	8–12

**Table 2:** Feature scaling information.

Feature	Scaler	Manual Range
Normalized Resonance Frequency	MinMaxScaler	[MIN_TEMP, MAX_TEMP]
Resonance Magnitude	MinMaxScaler	[−100, −30]
Resonance Phase	MinMaxScaler	[−180, 180]
Resonance Real	MinMaxScaler	[−0.003, 0.003]
Resonance Imaginary	MinMaxScaler	[−0.003, 0.003]
Temperature	MinMaxScaler	[MIN_TEMP, MAX_TEMP]

**Table 3:** Parameter ranges for Bayesian Optimization.

Hyper-Parameter	Min Value	Max Value	Value
Dropout	0.0	0.001	0.00018
Learning Rate	0.0009	0.0011	0.00097
Neuron Percentage	0.005	0.015	0.04269
Neuron Shrink	0.7	1.0	0.72329
Kernel Regularizer	0.0009	0.0011	0.00094

**Table 4:** Distribution of Experimental Datasets by Distance and Temperature Trend.

Distance (mm)	Rising Temp. Datasets	Falling Temp. Datasets	Total
200	3	4	7
350	4	3	7
500	4	4	8
750	2	2	4
900	1	1	2
**Total**	**14**	**14**	**28**

**Table 5:** Comparison of variance, $R^{2}$ score, and MAE across Linear Regression, NN, and LSTM models.

Model	Variance [°C]	$R^{2}$ Score [-]	Mean Absolute Error (MAE) [°C]
Linear Regression	1238.4	0.9494	5.7
Neural Network	1641.9	0.9661	5.4
LSTM	1377.0	0.9973	1.3

**Table 6:** Comparison of various temperature sensing technologies across key performance metrics.

Sensor Type	Method	Sensitivity	Max Interrogation Distance [m]	Accuracy	Response Time	Range [°C]
Patch Antenna (This Work)	Wireless	0.05–0.20 MHz/°C	0.75	0.997 $R^{2}$	∼10 s polling	20–170
Ceramic Patch Antenna [1]	Wireless	0.062–0.022 MHz/°C	0.75	0.92–0.97 $R^{2}$	∼Polls every 50 °C	25–1000
Thermocouple (Type S) [51]	Wired	~58.7 µV/°C	N/A	±1–2 °C	∼1 s	−50 to 1768
SAW Sensor [28]	Wireless	0.00762 MHz/°C	∼0.1	0.997 $R^{2}$	N/A	0 to 400
Infrared [52]	Optical	N/A	<5 (LoS)	±2 °C	∼50 Hz	−50 to 3000+

## Data Availability

The dataset is available on request from the authors, source code see https://github.com/OUIDEAS/LibreVNASniffer (accessed on 26 July 2024).
